# Emerging Hallmarks of Metabolic Reprogramming in Prostate Cancer

**DOI:** 10.3390/ijms24020910

**Published:** 2023-01-04

**Authors:** Francesco Lasorsa, Nicola Antonio di Meo, Monica Rutigliano, Matteo Ferro, Daniela Terracciano, Octavian Sabin Tataru, Michele Battaglia, Pasquale Ditonno, Giuseppe Lucarelli

**Affiliations:** 1 Urology, Andrology and Kidney Transplantation Unit, Department of Precision and Regenerative Medicine and Ionian Area, University of Bari “Aldo Moro”, 70124 Bari, Italy; 2 Division of Urology, European Institute of Oncology, IRCCS, 20141 Milan, Italy; 3 Department of Translational Medical Sciences, University of Naples “Federico II”, 80131 Naples, Italy; 4 The Institution Organizing University Doctoral Studies (I.O.S.U.D.), George Emil Palade University of Medicine, Pharmacy, Sciences and Technology, 540142 Târgu Mureș, Romania

**Keywords:** prostate cancer, metabolomics, androgen receptor, biomarkers, metastasis, castration resistance

## Abstract

Prostate cancer (PCa) is the most common male malignancy and the fifth leading cause of cancer death in men worldwide. Prostate cancer cells are characterized by a hybrid glycolytic/oxidative phosphorylation phenotype determined by androgen receptor signaling. An increased lipogenesis and cholesterogenesis have been described in PCa cells. Many studies have shown that enzymes involved in these pathways are overexpressed in PCa. Glutamine becomes an essential amino acid for PCa cells, and its metabolism is thought to become an attractive therapeutic target. A crosstalk between cancer and stromal cells occurs in the tumor microenvironment because of the release of different cytokines and growth factors and due to changes in the extracellular matrix. A deeper insight into the metabolic changes may be obtained by a multi-omic approach integrating genomics, transcriptomics, metabolomics, lipidomics, and radiomics data.

## 1. Introduction

Prostate cancer (PCa) is the most common male malignancy and the fifth leading cause of cancer death in men worldwide [1,2]. PCa may be suspected based on digital rectal examination (DRE) and/or prostate-specific antigen (PSA) levels; however, PSA levels may increase in many non-malignant clinical conditions. An early diagnosis of PCa reduces the cost for disease management and improves treatment efficacy and patients’ quality of life. In this scenario, the discovery of novel biomarkers that may increase the sensitivity and specificity for PCa diagnosis and prognosis (i.e., Prostate Cancer Antigen 3, the TMPRSS2-ERG gene fusion, Spondin 2, and circulating tumor cells) is of utmost importance [3,4,5,6]. Many studies have already marked the association between metabolic disorders and an increased risk of PCa, as well as for other urologic tumors [7,8]. Obesity is a well-known risk factor for PCa and an increased BMI is associated with aggressive disease. Obesity triggers a low-grade inflammation (metaflammation), which is thought to be a milestone for the pathophysiology of several chronic diseases as for cancer. As for diabetes, it was demonstrated that TNF and TLR signaling reduce insulin secretion and effects. This crosstalk between metabolism and the immune system has been shown to be evolutionarily conserved. Further future studies will be necessary to unravel molecular mechanisms linking aberrant metabolism and cancer [9,10]. Nevertheless, several previous studies have already stated that obese patients diagnosed with prostate cancer experience worse outcomes. They are at an increased risk of disease recurrence, exacerbated treatment-related adverse effects, the development of obesity-related comorbidities, earlier metastatic disease, and higher prostate cancer mortality. The increased inflammatory environment together with metabolic irregularities are commonly postulated, although physiological mechanisms linking obesity with patients’ poor prognosis remain unclear [11,12,13,14,15,16,17,18,19]. A recent study explored the metabolic and genetic signatures of obese individuals. The authors analysed metabolome perturbations in association with BMI and they found out that metabolite signatures (mBMI) better stratify patients’ health risk [20]. Metabolomics is referred to as the analysis of metabolic products in cells, tissues, organs, and the organism, which may be used to identify new biomarkers for cancer diagnosis and management [21]. The multi-omics approach including the study of cancer cell metabolism represents a powerful strategy for a better comprehension of cancer progression [22].

## 2. Metabolism of Normal and Tumor Prostate Cells

Prostate cells are characterized by an increased production of citrate and polyamines, which are physiological components of prostatic fluid. The high amount of citrate produced by prostate cells depends on a specific metabolic pathway not described in other human cells. In eukaryotic cells, glucose is transformed via glycolysis into pyruvate and then oxidized in acetyl-CoA in the mitochondria. Eventually, acetyl-CoA reacts with oxaloacetate, producing citrate, which enters the Krebs cycle. In prostate cells, zinc ion inhibits the mitochondrial enzyme aconitase ACO2 (first step of citrate oxidation), thus blocking the Krebs cycle and leading to citrate accumulation [23]. Citrate is then secreted in the semen where it regulates pH, ions homeostasis, and the coagulation/de-coagulation process. Citrate has high affinity for zinc, magnesium, and calcium, and it is the main regulator of ionized calcium concentration in semen. Variations in citrate secretion by prostate epithelial cells alter male fertility since calcium concentration affects sperm count, motility, morphology, and volume [24,25,26,27]. Zinc ion is a cofactor of several cell enzymes with structural, catalytic, and regulatory functions. Two different families of transporters have been identified: the ZIP (Zrt/IRT-like proteins) family that regulates zinc inflow from the extracellular zone, and the ZnT family (Zn transporter) that controls zinc outflow from the cell and ion redistribution into the mitochondria and lysosomes. These proteins are significantly downregulated in PCa [28,29,30,31,32,33,34,35,36]. As a result of the loss of zinc accumulation, a higher energy balance is obtained (via the Krebs cycle). As well as this role, zinc is involved in other biological processes such as cell division, intracellular signaling, apoptosis, cell invasion, and migration. Induced zinc accumulation in PCa cells leads to a marked inhibition of cell growth. The inhibitory effects was associated with increased levels of p21 [37]. Recent studies have demonstrated the influence of zinc in the expression of genes related to tumor growth, angiogenesis, and metastasis through the nuclear transcriptional factor NF-kB. The treatment of PCa with zinc was associated with a reduced expression of tumorigenic cytokines (VEGF, IL-6, IL-8 and MMP-9). Conversely, zinc depletion led to the increased expression of some genes (PKB/Akt, Mdm2 phosphorylation) and reduced levels of p53 and p21 [38,39]. Zinc accumulation has been associated with the mitochondrial release of cytochrome C, thus leading to the activation of caspase-9 and caspase-3 and the cleavage of poly(ADP-ribose)polymerase (intrinsic apoptosis pathway) [40,41]. Moreover, PCa cells pretreated with zinc showed a reduced expression of intercellular adhesion molecules (i.e., ICAM-1) and inactivation of Aminopeptidase N, with an impaired ability to invade the extracellular matrix [42,43,44].

## 3. Glucose Metabolism Reprogramming

Prostate cancer cells are characterized by a hybrid glycolytic/oxidative phosphorylation (OXPHOS) phenotype determined by androgen receptor (AR) signaling that may stimulate the AMPK-PGC1α cascade [45,46,47]. AMPK regulates the use of carbohydrates, lipids, and amino acids as energy sources, whereas peroxisome proliferator-activated receptor gamma coactivator 1-alpha (PGC1α) is a transcriptional coactivator playing a key role in mitochondrial biogenesis and function. The FGFR1 pathway involving lactate dehydrogenase (LDH) isoenzymes is one of the mechanisms responsible for the shift from OXPHOS to aerobic glycolysis [48]. The LDHA isoenzyme preferentially converts pyruvate to lactate, which can eventually be oxidized back to pyruvate by LDHB. Phosphorylated LDH isoform A and reduced expression of LDH isoform B (because of methylation of the gene promoter) may lead to the Warburg effect. At the early stages of cancer development, PCa cells are mainly independent of glucose since they use fructose as an alternative energy source: glucose transporter GLUT-1 is less expressed than fructose transporter (GLUT-5) [49]. Bader et al. showed that the mitochondrial pyruvate carrier (MPC) was transcriptionally regulated by AR. This carrier transports pyruvate into mitochondria and links cytosolic with mitochondrial metabolism, and it is increased in primary PCa and is associated with poor clinical outcomes [50]. Another important glucose pathway is the pentose phosphate pathway (PPP), which allows cells to obtain precursors for nucleotide synthesis and NADPH by using glucose-6-phosphate. In PCa cells, glucose-6-phosphate dehydrogenase (G6PDH), the rate-limiting reaction in PPP, is upregulated, and it was shown that AR signaling increases G6PDH, NADPH, and ribose synthesis [51,52,53]. Two genes (PRKAB1 and PFKFB4) were described by Ros et al. to be important for PCa cell survival [54]. PRKAB1 encodes for a regulatory subunit of AMP-activated kinase (AMPK), which turns off ATP-consuming pathways. LKB1 (an AMPK upstream kinase) expression was significantly reduced in high-grade PIN lesions and completely lost in adenocarcinomas [55]. The pivotal role of the LKB1-AMPK axis in controlling oncogenic processes depends on the interaction with the PI3K, mTOR, and MAPK pathways [56,57]. PFKFB4 encodes for 6-phosphofructo-2-kinase/fructose-2,6-biphosphatase 4 (an isoform of the glycolytic enzyme phosphofructokinase 2). Higher PFKFB4 mRNA levels were found in metastatic PCa compared with localized tumors because of the role played by this enzyme in controlling glycolysis and antioxidant production.

## 4. Lipid Metabolism Reprogramming

**Figure 1:**
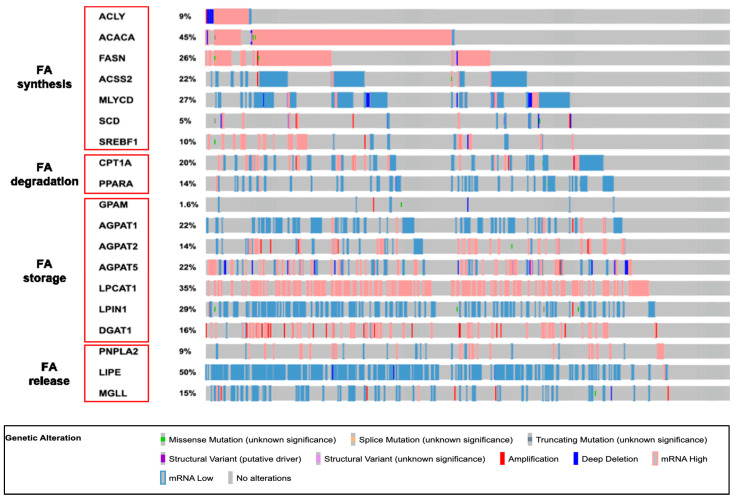
Oncoprints of fatty acid (FA) metabolism genes in the cancer genome atlas (TCGA, PanCancer Atlas) PCa patient cohort (PRAD). About 88% of the tumors from 489 patients in PRAD showed altered gene copy number or expression of FA metabolism-related genes. ACLY: ATP citrate lyase; ACACA: acetyl-CoA carboxylase alpha (ACC); FASN: fatty acid synthase; ACCS2: acyl-CoA synthetase short-chain family member 2; MLYCD: malonyl-CoA decarboxylase; SCD: stearoyl-CoA desaturase; SREBF1: sterol regulatory element-binding transcription factor 1 (SPREBP1); CPT1A: carnitine palmitoyltransferase 1A; PPARA: peroxisome proliferator-activated receptor alpha; GPAM: glycerol-3-phosphate acyltransferase; AGPAT: 1-acylglycerol-3-phosphate O-acyltransferase; LPCAT1: lysophosphatidylcholine acyltransferase 1; LPIN1: lipin 1; DGAT1: diacylglycerol O-acyltransferase 1; PNPLA2: patatin-like phospholipase domain containing 2; LIPE: lipase, hormone-sensitive; MGLL: monoglyceride lipase.

Lipids may be used as an energy source by the fatty acid (FA) β-oxidation in the mitochondria. Fatty acid translocase (FAT/CD36) is a main FA transporter. CD36 is frequently gained or amplificated in prostate cancer; this feature has been associated with patients’ poor prognosis. In addition, CD36 functions as a receptor capable of activating SRC family kinases, mitogen-activated protein kinases, and reactive oxygen species pathways. CD36 may recognise different ligands such as oxidized low-density lipoproteins, β-amyloid peptide, staphylococcus aureus-derived microbial diacylglycerides and lipoteichoic acid, and mycoplasma macrophage-activating lipopeptide-2. This feature may explain the oncogenic potential of CD36. It has been noted that blocking CD36 reduces fatty acid uptake from the tumor microenvironment, and reduces lipid biosynthesis and the oncogenic lipid signaling pathways, thus limiting cancer growth [58,59,60]. Fatty acid binding proteins (FABPs) regulate the intracellular trafficking of fatty acids. Metastatic progression of different cancers (including PCa) may also depend on FABPs. A cluster of FABP members (FABP4, FABP5, FABP8, FABP9, and FABP12) maps on the chromosome 8q21 region, which is commonly amplified in PCa metastases. It has been suggested that FABPs transfer FA to PPAR β/δ and γ. Downstream effectors of PPARs may enhance cancer epithelial–mesenchymal transition (EMT), angiogenesis, migration, and invasion [61,62,63,64]. A CoA-group is added to FA resulting in acyl-CoA, which will be oxidized into acetyl-CoA in the mitochondria. Eventually, acetyl-CoA will enter the TCA cycle. Fatty acids are part of monoacylglycerols, diacylglycerols (DAG), triacylglycerols (triglycerides), sterol esters, and membrane phospholipids. Cholesterol synthesis demands acetyl-CoA to begin; the rate-limiting reaction is catalyzed by HMG-CoA reductase. As well as fatty acids’ de novo synthesis, they are also derived from adipose tissue lipolysis or breakdown of triglycerides contained in circulating chylomicrons and lipoproteins. An increased lipogenesis and cholesterogenesis have been described in PCa cells (Figure 1) [65].

Many studies have shown that the enzymes involved in these pathways are overexpressed in PCa. ATP-citrate lyase (ACLY), acetyl-CoA carboxylase (ACC), fatty acid synthase (FASN), stearoyl-CoA desaturase-1 (SCD1), 3-hydroxy-3-methyl-glutaryl-CoA reductase (HMGCR), and squalene epoxidase (SQLE) are overexpressed in cancer cells and are under the transcriptional control of the AR. Sterol regulatory element-binding proteins (SREBPs- three isoforms 1a, 1c and 2) are transcription factors that bind sterol regulatory elements (SRE) in the promoter region of genes involved in fatty acid, cholesterol, and lipid metabolism (HMG-CoA synthase, HMG-CoA reductase, FASN, SCD1, and LDLR) [66]. To be activated, a SREBP demands the cleavage from the endoplasmic reticulum (ER) membrane in a two-step process in the Golgi apparatus by the SREBP cleavage-activating protein (SCAP). SREBP2 isoform regulates cholesterol metabolism by inducing the mevalonate pathway, while SREBP1 activates fatty acid synthesis [67]. The SREBP activity depends on the sterol intracellular concentration since the SCAP-SREBP complex is retained at the rough ER when cholesterol is available. The molecular mechanism of this sterol-sensing system is still unclear. Other alternative pathways have been described with SREBP activation such as TNF-α and mTORC [68,69]. The loss of PTEN (a frequently observed genomic anomaly) and the upregulation of PI3K/AKT/mTOR lead to the activation of SREBPs. In addition, SREBP1 has been described as being involved in PCa cell proliferation, migration, and invasion by activating lipogenesis and through an increased production of reactive oxygen species and NADPH oxidase 5 expression. AR activates the expression of SCAP, and SREBP1 increases the expression of the AR. So, the AR-SREBP1 axis forms a self-regulating loop to keep a continuous gene expression [70]. In castration-resistant cancer cell lines, decreased tumor growth, increased apoptosis, altered lipidome, decreased lipid storage, and AR and AR-V7 expression have been observed after inhibiting FASN activity, thus highlighting the role that this enzyme may play in PCa progression [71]. The aberrant accumulation of esterified cholesterol has been observed in lipid droplets within high-grade and metastatic human PCa, but is not detectable in normal prostate cells. The abundance of cholesterol is related to the upregulated producing pathways and uptake from the circulation, whereas sterol efflux is downregulated (ATP binding cassette transporter A1 or G1 are cell transport proteins) [72]. Statins (inhibitors of HMG-CoA reductase) reduce PCa cells’ growth, invasion, and migration, and induce apoptosis. Increased expression of HMG-CoA reductase has been associated with poor prognosis; in addition, a higher expression of this enzyme has been noted in enzalutamide-resistant PCa cells, and its knockout can restore enzalutamide sensitivity. The combination of simvastatin and enzalutamide decreased PCa cell growth in both in vivo and in vitro models. On the other hand, simvastatin alone or in combination with enzalutamide lowered AR expression in enzalutamide-resistant cancer cells [73,74,75,76,77].

Lipidomic analysis revealed that monounsaturated fatty acid and polyunsaturated fatty acid levels overcome free saturated fatty acids. Overexpression of Δ-Enoyl-CoA Delta Isomerase 1 (EC1), a key enzyme of β-oxidation, has been associated with the biochemical recurrence of PCa [78]. In turn, the inhibition of this enzyme reduced cell growth. Furthermore, 2,4 dienoyl-CoA reductase (DECR1, involved in polyunsaturated fatty acyl-CoA oxidation) is more expressed in castration-resistant tumors. Knockdown of DECR1 has been shown to reduce proliferation, migration, and treatment resistance [79].

High levels of phosphocholine, phosphoethanolamine, and glycerophospholipids have been observed in PCa; these compounds are consistent with membrane remodeling and cellular proliferation processes [80]. The “cholinic phenotype” is referred to as the overexpression of choline kinase alpha (CHKA) and phosphocholine (PCho) levels in different types of cancers, including PCa. Two separate genes (CHKA and CHKB) encode for three isoforms, CHKA-1, CHKA-2, and CHKB, which are active in homodimeric, heterodimeric, and oligomeric forms. High levels of CHKA-1/2 have been observed in PCa and in several human malignancies (breast, lung, ovarian, endometrial, colorectal, bladder cancers, osteosarcoma, and T-cell lymphoma). PCho is the main source for phosphatidylcholine (PC-Kennedy pathway), which is the main phospholipid in eukaryotic membranes with other associated functions such as cholesterol transport support, a substrate to produce second messengers, and a cofactor for several enzymes [81,82]. CHK is also involved in sphingomyelin synthesis, another essential membrane phospholipid [83]. The working hypothesis is that AR activity and PCa growth may be negatively influenced by reducing CHKA levels. Blocking this kinase may allow cancer cells to activate alternative pathways for phospholipids synthesis that have toxic effects and may lead to cell destruction. Different effective CHKA inhibitors have been developed in clinical trials, but they soon manifested elevated toxicity.

Sphingomyelinases degrade sphingomyelin (SM) into ceramide and phosphocholine. Sphingomyelin synthases (SGMSs) use phosphatidylcholine and ceramide to form SM and diacylglycerol (DAG) [84]. High levels of SM ensure cell survival, proliferation, migration, and inflammation, whereas high levels of ceramide provoke cell cycle arrest. SGMS and ceramide kinase (CERK) regulate sphingolipid homeostasis. In cancer cells, SGMS activity keeps low levels of ceramide; moreover, ceramide 1-phosphate (produced by CERK-dependent phosphorylation of ceramide) plays an important role in cell proliferation and migration and in cancer aggressiveness [85]. Metastasis-associated lung adenocarcinoma transcript-1 (MALAT1) is a long non-coding RNA highly expressed in various malignancies such as bladder, lung, and prostate cancer. It has been hypothesized that MALAT1 interferes with gene expression at a post-transcriptional level mediated by micro-RNAs. It suppresses miR-140 in PCa cells [86]. MALAT1 depletion induces metabolic reprogramming of cancer cells toward a more glycolytic phenotype. Reduced energy balance does not support cell growth adequately. It has been described that CHKA and CERK are targets of MALAT1 as well as MALAT1 modulating CHKA expression, PCho, and glutathione content in PCa cells [87,88]. Since choline synthesis is a crucial event for cancer cells, MALAT1 targeting downregulates CHKA and CERK activity; this effect seems to overlap the activity of CHKA inhibitors. Heat shock proteins (HSPs), which are upregulated in PCa, act as chaperones by binding the ligand-binding domain (LBD) of AR, promoting its stability, folding, and activation. It has been shown that CHKA acts as a co-chaperone reinforcing AR signaling by binding to its LBD. Hence, a feed-forward AR-CHKA signaling loop exists. This reinforces the evidence of CHKA as a marker of tumor progression and a potential target for PCa [89].

Other metabolic intermediates of de novo lipogenesis, whose concentrations are significantly increased in PCa cells, are diacylglycerol (DAG), phosphatidic acid (PA), 6-cholesteryl ester (CE), sphingosine 1-phosphate (S1P), and lysophosphatidic acid (LPA) [90].

## 5. Amino Acid Metabolism Reprogramming

Glutamine represents the main source for the Krebs cycle and lipogenesis intermediates. It enters the cells through the solute carrier (SLC) group of transporters or via micropinocytosis from the surrounding microenvironment [91,92,93]. Glutamine is transformed into glutamate by glutaminase GLS1 then turned into α-ketoglutarate (α-KG) by glutamate dehydrogenase (GDH) or glutamic oxaloacetic transaminase (GOT). Glutamine is incorporated into the TCA cycle, and it is a building block for glutathione synthesis. α-KG serves as a cofactor for Fe (II)-α-KG dioxygenase, which contributes to DNA demethylation and for Jumonji-domain-containing histone demethylases as well [94,95,96]. An increased expression of glutamine transporter ASCT2 (SLC1A5) and of glutaminase GLS1 has been observed in PCa cells, so glutamine becomes an essential amino acid. These events have been associated with AR, MYC, and mTOR pathways’ activation [97]. Moreover, glutamine functions as a nitrogen donor for amino acids, nucleotides, and other important metabolites for tumor growth. Because of its essential role in prostate cancer progression, glutamine metabolism is thought to become an attractive therapeutic target [98,99]. Glutamate may also be obtained by cancer cells by N-acetyl-aspartyl-glutamate (NAAG) when other ways are limited. Recent studies have explored the relationship between plasma NAAG concentrations and tumor sizes for different cancer, whereas glutamate levels seem to correlate with the Gleason score [100,101]. Tryptophan (TRP) accumulates in cancer compared to normal prostate tissue. Indoleamine-2,3-dioxygenase (IDO1) and tryptophan 2,3-dioxygenase (TDO) mediate the conversion of TRP into KYN. IDO1 expression is under IFN-γ and TNF-α control. The role of KYN seems to be mediated by the interaction with the cytoplasmic aryl hydrocarbon receptor (AhR) in immune and cancer cells. After binding with KYN, AhR moves into the nucleus promoting target genes involved in tumor cell migration and immune evasion. The TRP/KYN pathway has already been described in both renal and bladder cancers [102,103]. Sarcosine is an N-methyl derivative of glycine whose expression increases during the progression from benign tissue, through localization, to metastatic cancer. Glycine-N-methyltransferase (GNMT) catalyzes the transfer of the methyl group from S-adenosyl methionine (SAM) to glycine. Recently, higher expression of GNMT have been described in PCa cells compared to normal tissues, and higher enzyme levels have been associated with lower disease-free survival rates [104]. A previous study showed that serum sarcosine had a higher predictive value than PSA in patients with PSA <4 ng/mL. At the same time, low-/intermediate-/high-grade cancers were positively associated with sarcosine levels [105,106].

## 6. Metabolic Crosstalk in Prostate Cancer Microenvironment

**Figure 2:**
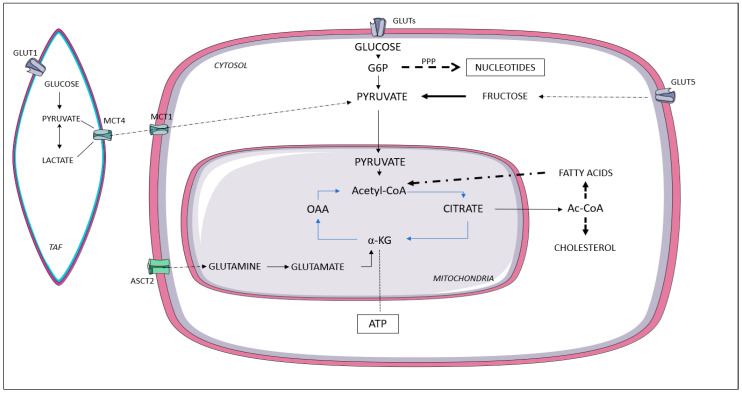
Overview of prostate cancer cell metabolism and its crosstalk with stromal components. TAF: tumor-associated fibroblast; MCT4: monocarboxylate transporter 4; MCT1: monocarboxylate transporter 1; GLUT: glucose transporter; G6P: glucose 6-phosphate; PPP: pentose phosphate pathway; α-KG: α-ketoglutarate; OAA: oxaloacetate; Ac-CoA: acyl-CoA; ASCT2 (SLC1A5): neutral amino acid transporter; ATP: adenosine triphosphate.

A crosstalk between cancer and stromal cells occurs in the tumor microenvironment because of the release of different cytokines and growth factors and due to changes in the extracellular matrix (Figure 2).

This remodulation of the crosstalk ensures not only cancer cells’ proliferation in a hostile environment but also their ability to infiltrate the ECM and metastasize. Cancer-associated fibroblasts (CAFs) and tumor-associated macrophages (TAMs) actively interact with cancer cells. As well as their ability to induce epithelial–mesenchymal transition and stem-like characteristics in PCa, CAFs induce reciprocal metabolic reprogramming. Indeed, glucose transporter GLUT1 is more expressed in CAFs. Once CAFs differentiate into myofibroblasts, they can secrete lactate and pyruvate (through monocarboxylate transporter-MCT4). These metabolites can be taken up by epithelial cancer cells (through the MCT1 transporter) and then incorporated into the Krebs cycle. Within cancer cells, lactate activates the SIRT1/PGC-1α axis, increasing mitochondrial mass and activity. Mitochondrial transfer between CAFs and cancer cells has recently been discovered [107]. This interaction makes cancer cells independent from glucose consumption while becoming dependent on lactate consumption for anabolic pathways and cell proliferation. This metabolic reprogramming between CAFs and PCa cells has been referred to as the “Reverse Warburg Effect” by Pavlides et al. In addition, CAFs provide high levels of glutamine by activated oncogenic Ras [108,109,110]. Adipocytes represent another class of stromal cells with a well-known crosstalk with PCa. Obesity and high visceral fat may increase the risk of PCa progression to metastatic disease probably because of the excess in dietary fatty acids, alterations in the insulin-IGF-1 axis, and higher levels of pro-inflammatory cytokines [111]. Several mechanisms have been suggested to explain the obesity–cancer association such as the presence of cancer-associated adipocytes, adipokines, obesity-related inflammatory cytokine production, and alteration in sex hormone metabolism. Phenotypical changes have been described in tumor-surrounding adipocytes: as well as the downregulation of adipocyte markers and reduced lipid storage, the overproduction of pro-inflammatory cytokines and extracellular matrix-related molecules has been noted [112]. Cell co-culture studies demonstrated that PCa cells store fatty acid from surrounding adipocytes. Indeed, increased expression of fatty acid transporters such as CD36 and FATP5 has been described in some PCa cell lines (PC-3 and LNCaP cells). In addition, in tumor cells, free fatty acids induce oxidative stress through NADPH oxidase 5 (NOX5); the increased ROS production contributes to the tumor cell invasion by activating the HIF1-MMP14 pathway. The increased thickness of periprostatic adipose tissue (PPAT) has been associated with PCa progression and the presence of high-grade disease. Contact with PPAT may promote progression in PCa with extraprostatic extension, and a decreased biochemical recurrence-free survival has been speculated after radical prostatectomy when compared to tumors not invading outside the prostate capsule [113].

Altuna-Coy et al. observed changes in linoleic acid metabolism. It may be transformed into 9- and 13-hydroxyoctadecadienoic acid (9- and 13-HODE) and 9- and 13-oxo-octadecadienoic acid (9- and 13-oxoODE). 13-oxoODE has been stated to be an endogenous ligand of PPARG with anti-inflammatory properties. They found reduced concentrations of 13-oxoODE/9-oxoODE in high-risk PPAT and reduced expression of PPARG and ADIPOQ genes. Reduced expression of PPARG (inhibitor of NF-kB) may explain the increased levels of pro-inflammatory cytokines (IL-6, TNF-α, IL-1B) in the aggressive PCa-related PPAT [114]. Finley et al. noted that adipokines’ concentrations in PPAT were more elevated than serum levels. In particular high levels of IL-6 were found in PPAT together with the increased phosphorylation of STAT3 in high-grade tumors [115]. Moreover, one of the chemokines involved in PCa progression in a mouse model is CXCL12/SDF-1. Saha et al. demonstrated that CXCL12 and its receptors CXCR4 and CXCR7 activate many oncogenic signaling pathways including STAT3, NFkB, and MAPK [116]. Laurent et al. described the role of CCL7, another chemokine secreted by adipocytes in PPAT. Interacting with CCR3 (overexpressed in PCa cells), CCL7 diffuses from the PPAT to the peripheral zone of the prostate, promoting cancer extraprostatic extension and local dissemination [117]. Leptin, a cytokine secreted by adipocytes, promotes MCT4 and its chaperone CD147 expression in cancer cells [118]. According to the density and location of tumor-infiltrating CD8+ T cells, and the presence of immunosuppressive Foxp3+ regulatory T cells and CD11b+ myeloid-derived suppressor cells (MDSC), cancers may be classified as immunologically “hot” or “cold”. Prostate cancers are known to be immunologically cold because of low cytotoxic T-cell infiltration. Tumor-associated macrophages (TAM) regulate angiogenesis, and tumor cells’ proliferation and dissemination. TAM polarization from the M1 phenotype toward the M2 phenotype relates with poor prognosis since they release anti-inflammatory cytokines (IL-10 and TGF-β), thus blocking cytotoxic CD8+ T-cell activity [119,120].

## 7. Metabolic Gene Alterations

The most frequently observed genomic alteration is PTEN (phosphatase and tensin homolog tumor suppressor) loss on chromosome 10 [121]. PTEN is a direct antagonist of PI3K, which has several important downstream effector molecules, including Ser/Thr protein kinase AKT/PKB, which finally activates the mammalian target of rapamycin (mTOR) [122]. Another possible genetic aberration is the gain of MYC. According to their genetics, two metabolic phenotypes have been described: phospho-AKT^high^/MYC^low^ versus phospho-AKT^low^/MYC^high^. AKT signaling activation has been associated with enhanced aerobic glycolysis (Warburg effect), the pentose phosphate pathway, and fructose metabolism; on the other hand, MYC overexpression has been related to the increased expression of glutaminase, dysregulated lipid metabolism, and reduced expression of some glycolytic enzymes [123,124,125]. However, both metabolic phenotypes have been associated with the increased expression of fatty acid synthase (FASN) [126]. Even if the clinical implications are still unknown, the characterization of these cellular subtypes may have diagnostic and therapeutic roles: 18F-FDG PET has a better diagnostic performance in AKT^high^/MYC^low^ PCa. SOX2 is a transcriptional factor with a pivotal role in stem cells’ pluripotency. Its expression has been described in different human malignancies including prostate cancer. First, SOX2 promotes mitochondrial biogenesis and enhances glycolysis, oxidative phosphorylation, purines, pyrimidines, amino acids’ metabolism, and the pentose phosphate pathway. At the same time, SOX2 has different gene targets not typically observed in human embryonic stem cells (i.e., BCL2, EZH2, FGFR3, FOXA1, KRAS, MET, etc.). Nevertheless, its mechanism of action remains unclear. It has been suggested that SOX2 may be involved in driving metastatic survival and growth, and in enabling easier adaptation to the new metastatic microenvironment. Furthermore, it has been shown to be associated with resistance to AR antagonist enzalutamide [127]. 

Ongoing insights are investigating autophagy and its crucial role in cancer biology. It is a homeostatic process for degradation through the lysosomal system of either older proteins or dysfunctional cytoplasmic organelles [128]. It contributes to the restoration of energy balance, especially during energy deprivation. In addition, autophagy aims to regulate cellular mass, to distribute organelles properly, and to remove harmful compounds. Depending on the cellular context, it may play either a protective or a detrimental role for PCa survival. mTORC plays a pivotal role in autophagy regulation [129]. Different genes related to this process involved in PCa carcinogenesis have been identified upstream of mTORC activity. STK11 encodes for LKB1, a tumor suppressor serine–threonine kinase whose main effector is AMPK. It has been described that LKB1 expression reduces throughout PCa progression from normal to neoplastic tissue [130,131]. Calcium/calmodulin-dependent protein kinase kinase 2 (CaMKK2) is another essential element for autophagic processes, which has been shown to be a target gene of AR [132]. Tumor necrosis factor-related apoptosis-inducing ligand (TRAIL) also triggers autophagy in PCa cells [133]. Autophagy may be thought of as a response mechanism to stressors for PCa including chemotherapy and castration therapy. In turn, autophagy itself may promote castration resistance. Cell death may be enhanced under anticancer treatment by inhibiting autophagic pathways. Further studies could elucidate the role of autophagy modulators to improve therapeutic strategies.

## 8. Conclusions

Blood, urine, semen, and tissue samples continue to be investigated for the identification of novel biomarkers that might become eligible for PCa diagnosis with a better performance than PSA. At the same time, new markers might be used for selecting candidates for active surveillance. To achieve these goals, algorithms of machine learning (ML), a branch of artificial intelligence (AI), are currently being developed and trained. Deep learning (DL) is a recent field of ML that has already shown superior problem-solving capabilities by using neural networks [134]. In the near future, by integrating different metabolomic datasets and improving AI techniques, new biomarkers for earlier cancer diagnosis might be identified. Prostate cancer cells face important reprogramming of different metabolic pathways. PCa cells and tumor microenvironment (TME) components are known to affect their metabolism mutually. A better comprehension of the relationship between cancer cells and TME might help novel effective therapies to be designed, which might overcome castration resistance disease. In association with standard therapies, targeting these altered metabolic pathways may represent future tools to improve prostate cancer treatments. Drugs targeting specific metabolic pathways are listed in Table 1. A deeper insight into the metabolic changes may be obtained by a multi-omic approach integrating genomics, transcriptomics, metabolomics, lipidomics, and radiomics data [135,136].

## Figures and Tables

**Table 1:** Cancer drugs targeting metabolic pathways that are approved, or under investigation in clinical trials.

Metabolic Pathway	Target	Drug
Glycolysis	GLUT1	WZB117, BAY-876
	Hexokinase	2-Deoxyglucose
	Pyruvate Kinase M2 (PKM2)	TEPP-46
	Lactate dehydrogenase A (LDHA)	Quinoline, 3-sulfonamides, FX11, PSTMB
	Monocarboxylate transporter 1 (MCT1)	AZD3965
Amino acid metabolism	Glutaminase 1 (GLS1)	CB-839, IPN60090
	ASCT2 (SLC1A5)	GPNA
	Multiple targets	JHU-083, DRP-104
	Phosphoglycerate dehydrogenase (PHGDH)	CBR-5884, NCT-503
	Indoleamine-2,3-dioxygenase-1 (IDO1)	Epacadostat, indoximod
	Circulating asparagine	L-Asparaginase
	Large neutral amino acid transporter (LAT1)	JPH203
Mitochondrial metabolism	Pyruvate dehydrogenase (PDH), α-ketoglutarate dehydrogenase	CPI-613
	Electron transport chain complex 1	Metformin, IACS-010759, IM156
Lipid metabolism	ATP-citrate lyase (ACLY)	SB-204990
	Acetyl-CoA carboxylase (ACC)	Soraphen-A
	fatty acid synthase (FASN)	TVB-2640
Enzymes mutated in cancer	Mutant isocitrate dehydrogenase 1 (IDH1)	Ivosidenib, IDH305, BAY1436032, LY3410738, FT-2102
	Mutant isocitrate dehydrogenase 2 (IDH2)	Enasidenib
Nucleic acid synthesis	Thymidylate synthase (TS)	5-Fluorouracil, capecitabine, pemetrexed, raltitrexed
	Dihydrofolate reductase (DHFR)	Methotrexate, pemetrexed
	Glycinamide ribonucleotide formyltransferase (GARFT)	Pemetrexed
	Dihydroorotate dehydrogenase (DHODH)	Brequinar, leflunomide
	Ribonucleotide reductase (RNR)	Gemcitabine, clofarabine, fludarabine, cladribine, cytarabine
	5-Phosphoribosyl-1-pyro-phosphatase (PRPP) amidotransferase	Mercaptopurine, thioguanine

## Data Availability

Not applicable.
